# Criminalization of scientific misconduct

**DOI:** 10.1007/s11019-018-9865-7

**Published:** 2018-08-28

**Authors:** William Bülow, Gert Helgesson

**Affiliations:** 1 Department of Philosophy, Stockholm University, Stockholm, Sweden; 2 Stockholm Centre for Healthcare Ethics (CHE), Department of Learning, Informatics, Management and Ethics, Karolinska Institutet, Stockholm, Sweden

**Keywords:** Criminalization, Fabrication, Falsification, Plagiarism, Scientific misconduct

## Abstract

This paper discusses the criminalization of scientific misconduct, as discussed and defended in the bioethics literature. In doing so it argues against the claim that fabrication, falsification and plagiarism (FFP) together identify the most serious forms of misconduct, which hence ought to be criminalized, whereas other forms of misconduct should not. Drawing the line strictly at FFP is problematic both in terms of what is included and what is excluded. It is also argued that the criminalization of scientific misconduct, despite its anticipated benefits, is at risk of giving the false impression that dubious practices falling outside the legal regulation “do not count”. Some doubts are also raised concerning whether criminalization of the most serious forms of misconduct will lower the burdens for universities or successfully increase research integrity. Rather, with or without criminalization, other measures must be taken and are probably more important in order to foster a more healthy research environment.

## Introduction

Scientific misconduct and fraud is a prevailing problem in science and threatens to undermine integrity, credibility and objectivity in research (Fanelli 2009; Pickett and Roche 2018). It also risks undermining trust, both among researchers and the general public (Shamoo and Resnik 2009; Hansson 2011; Resnik 2014). It is therefore important to consider the possible means of countering fraud and misconduct in research. The perhaps most drastic suggestion is that at least the most serious forms of fraud and misconduct, such as fabrication and falsification, should be criminalized and, when it has occurred, punished (Redman and Caplan 2005, 2015; Sovacool 2005; Bhutta and Crane 2014; Pickett and Roche 2018). By criminalization we here mean the decision of making some action a criminal offense for which one may merit criminal punishment, such as fines, community service or even incarceration.

The need for criminalization of scientific misconduct has been defended in the bioethics literature (see e.g., Bhutta and Crane 2014; Redman and Caplan 2005, 2015; Sovacool 2005; Pickett and Roche 2018). However, from a philosophical point of view, the decision to criminalize a particular action stands in need of a moral justification. Criminal punishment, such as imprisonment, involves either intentional harm or the intentional deprivation of some good, such as liberty. It also has negative consequences for family members and those socially, economically and emotionally dependent on the person being punished. Granted that there is a *prima facie* duty not to intentionally harm others, a moral justification for criminalizing a certain behavior, and thereby allowing for the imposition of punishment, is therefore required.

Among those who defend the need of criminalizing serious scientific misconduct, the assumption is often made that fabrication, falsification and plagiarism (FFP) are the most serious forms of misconduct (Bhutta and Crane 2014; Sovacool 2005). In line with this assumption, it has been argued that these are the types of misconduct that should be criminalized, whereas other forms of misconduct, such as selective presentation of results, sloppiness and inappropriate use of statistics, are explicitly excluded (Sovacool 2005). Others reject this idea, arguing that serious misconduct that should be criminalized includes more than FFP (Redman and Caplan 2005; Pickett and Roche 2018).

In this paper, we will raise a number of concerns with regards to proposals of both kinds. The ambition is not to give the final answer to whether or not scientific misconduct should be criminalized and, if it should, how the details of such law should be construed, but rather to provide some first steps by pointing out difficulties that need to be considered before a decision can be made. In particular, we argue that given a tentative moral justification for criminalizing research fraud and scientific misconduct, a law targeting only or primarily fabrication, falsification and plagiarism is both *too inclusive* and *too exclusive*; that is, it would include acts that arguably should not be criminalized and exclude behavior that arguably should be, at least if fabrication or falsification is so. Even if drawing the line at FFP does not work, it can be difficult to decide which instances of scientific misconduct ought to be criminalized.

But even if it is possible to draw the line in a non-arbitrary way, we argue that if the aim of criminalization is to protect integrity and objectivity in research, it is not clear that criminalizing the most serious forms of scientific misconduct will accomplish all that would often be expected of such a law. On the contrary, it may cause problems in its own. The criminalization of scientific misconduct, despite its anticipated benefits, is at risk of giving the false impression that dubious practices that fall outside the legal regulation “do not count”. It is also far from certain that criminalization of the most serious forms of misconduct will lower the burdens for universities or successfully increase research integrity. Rather, with or without criminalization, other measures must be taken and are probably more important in order to foster a more healthy research environment. However, before we develop our arguments in greater detail, we will first look at the possibility of justifying the criminalization of scientific misconduct in the first place.

## The moral basis for criminalization of scientific misconduct

Criminalization is an important topic in legal philosophy and the philosophy of criminal law, and substantial discussion has been devoted to the appropriate scope and use of criminal law (see e.g., Feinberg 1984; Husak 2008; Moore 2009; Duff 2014). Despite this, bioethical discussions on whether to criminalize scientific misconduct rarely engage with this literature, at least as far as we know. Instead claims are sometimes made among those who defend criminalization that many instances of research fraud are similar to other types of criminalized behavior, such as financial fraud or theft, and that scientific fraud should therefore not be treated any differently (see e.g., Redman and Caplan 2015). Arguments are also often made that sanctions are required for the sake of deterring potential fraudsters (e.g., Sovacool 2005; Redman and Caplan 2005, 2015; Bhutta and Crane 2014). Underlying each of these arguments is the concern that scientific misconduct has negative effects on research integrity. Also the negative impact it might have on research participants and society as a whole is emphasized.

Arguments by analogy are often weak. In particular, arguments of the kind that “B is justified because B is similar to A in the relevant sense, which is justified” leave the core question unresolved, since it does not show either that B or A is justified. Although comparing research misconduct to behavior that is criminal has strong rhetorical effect, the relevant question is whether they share relevant moral properties and wrong-making features. And even if they do, it is not clear that sharing relevant wrong-making features is enough for criminalization. For example, not all forms of dishonest behavior are considered to be criminal, although they resemble other forms of fraudulent and dishonest behavior already criminalized. Hence, we better leave the analogies aside and address directly what is the appropriate scope and use of criminal law.

Without adhering to a specific normative theory of criminalization, we assert that for an act or behavior to be criminalized it must fulfill the following three criteria:

1. The act to be criminalized should cause or threaten to cause* non-trivial harm* to others
2. The act should be* wrongful*
3. The law criminalizing the act should foster or promote an important value that is independent of the law

Our first criterion is a version of the Millian harm principle, according to which the state is allowed to interfere with an individual’s liberty and autonomy for the sake of preventing harm to others, whereas harmless immorality (if there is such a thing) is not a sufficient ground for either criminalization or state interference (Mill 1977). However, in order to be criminalized, and to ultimately merit criminal punishment, the act or behavior must also satisfy the *wrongfulness constraint*. In other words, the offense must constitute a wrong in a relevant sense, which is the second criterion. What qualifies as wrongful is, of course, open for debate. Following Douglas Husak (2009), we assert that the content of the wrongfulness constraint should be derived largely from moral philosophy. At the very least agents who intentionally and willingly do something that causes or threatens to cause non-trivial harm to others, without there being a sufficient moral reason to do so, are doing something wrongful. Grave negligence, of some sorts, would also qualify as being wrongful, as when there is a duty involved, for instance a duty not to expose research participants to serious risks of harm.

The claim that one only ought to criminalize wrongful acts which cause, or threaten to cause non-trivial harm, are often assumed in the context of criminal law, and it has been argued that this restriction provides *internal* constraints on criminalization (see e.g., Husak 2008). As Husak points out, the fact that an offense should involve non-trivial harm is asserted in defenses, such as the defense of *de minimis*, which suggests that a defendant’s conduct did not actually cause or threaten to cause the sort of harm which the law sought to prevent, or did so only to an extent that is too trivial to merit any condemnation of conviction (Husak 2008, p. 67). Similarly, common excuses, such as the claim that the defendant committed the offense under duress, suggests that he is less blameworthy (or perhaps not blameworthy at all) than if there had been no excuse. This asserts, however, that the original criminal offense is wrongful. Otherwise excuses of this kind would not make any sense (Husak 2008, p. 73). One might also add that since punishment is a matter of expressing blame or censure, it is ultimately concerned with wrongdoing (see e.g., Duff 2014).

As we discuss in the following section, criminalization should not only target wrongful acts that cause or threaten to cause non-trivial harm, but should also serve compelling instrumental purposes and promote an important value that is independent of the law. However, if we first focus on the non-trivial harm and wrongfulness criteria alone, it is *prima facie* plausible that serious forms of scientific misconduct may be criminalized. Fabrication and falsification, for example, are arguably both wrongful and potentially harmful (Resnik 2014). In medical science, they may be harmful due to the consequences of being misleading about such things as treatment effects and patient safety. Besides its direct harm, it is also important to note that fraud can jeopardize the goods that may come out of science. Scientific misconduct can also easily undermine the public’s trust in research, as it often attracts massive attention in public discourse (Resnik 2014; Hansson 2011). As Sven Ove Hansson has pointed out, this implies that research requires strict ethical standards in order to ensure public trust and minimize the risk that essential scientific activities, including clinical trials and animal experimentation, become difficult or impossible to perform (Hansson 2011).

That wrongful acts causing non-trivial harm may be criminalized does not mean that they must be. There may be good reasons why criminalization should be resisted even if these two criteria hold. While some philosophers may be inclined to say that the law should track morality, and that the fact that an act constitutes a serious moral wrong is in itself a reason to criminalize it, a more reasonable position, we think, is that having the law should also foster or promote important values that are independent of the law, such as preventing wrongdoing or promoting distributive or retributive justice (Segev 2017). In the next section, we discuss the various values and ends that the criminalization of scientific misconduct could promote.

## The rationale for criminalization of scientific misconduct

The question whether criminalizing scientific misconduct helps promoting important values has been subject to discussion in the bioethical literature. The main argument in favor of criminalizing scientific misconduct is that it is needed for the sake of protecting research integrity as well as research participants and the public at large (Bhutta and Crane 2014; Redman and Caplan 2005, 2015; Sovacool 2005). For instance, Sovacool (2005) argues that stricter penalties could help *deter* intentional acts of scientific misconduct, such as fabrication, falsification and plagiarism. In combination with better protection for whistle-blowers it could also encourage colleges to report instances of misconduct and give raise to institutional reform with the aim of fostering integrity and honesty in the scientific community (Sovacool 2005). Similarly, Redman and Caplan (2005) suggest that criminal sanction for the most egregious cases of scientific misconduct might sufficiently raise the stakes to serve as a deterrent. In support of this claim they point out that if the maximum penalty for serious misconduct is no worse for junior researchers than the routine “penalty” for not publishing in high-ranked journals, this is not sufficient as a deterrent given the benefits fraud may have for the individual who gets away with it (Redman and Caplan 2005). Therefore, being denied research funding or lose the possibility of securing a future career in academia is not enough as a deterrent. If they are right, this means that criminalization of serious forms of scientific conduct can be morally justified.

Many of the arguments we have seen in defense of criminalizing scientific misconduct focus on deterrence as the overall justificatory aim of punishment. In contrast, other penal aims are not mentioned—or at least not explicitly so. It is therefore worth pointing out that arguments given in support of criminalizing scientific misconduct may also find support in the idea of retributive justice or from expressivist theories of punishment. According to standard retributivist theories, it is intrinsically morally good that wrongdoers get what they deserve, which is to suffer a punishment proportionate to the wrongfulness of their action (Walen 2016). In relation to scientific misconduct, some may argue that merely being denied opportunity to apply for research funding or being able to secure an academic position is not a proportionate punishment for deliberate scientific misconduct, especially since these are consequences that an unsuccessful junior researcher would face anyhow. A similar conclusion can be made on behalf of expressivist theories of punishment, in which the aim of punishment is to communicate an appropriate moral condemnation or public denunciation of the wrongdoing (Wringe 2016). Insofar as the lack of formal punishment amounts to a failure to provide an appropriate moral disapproval, the expressivist could also favor criminalization of serious forms of scientific misconduct in order to be able to communicate appropriate moral blame or public disapproval (Pickett and Roche 2018).

Besides penal aims, defenders of criminalizing scientific misconduct hold that the basic due process that would follow from criminalization is necessary in order to ensure a fair and balanced misconduct investigation (Sovacool 2005; Redman and Caplan 2005). Another potential benefit of criminalization is that it would take away the burden associated with such investigations from the universities, but also increase the legitimacy and efficiency of such investigations (Sovacool 2005).

In contrast to the above, others have argued that there are other, non-punitive ways, in which to deal with the prevalence of scientific misconduct besides criminalization. For instance, Birgitta Forsman has argued that we need to copy the safety thinking of aviation, introduce a system for “deviation reports” and focus on measures aimed at eliminating the risk for “accidents” (Forsman 2009). One of us has argued elsewhere that there is an essential lack of analogy between aviation safety and reduction of scientific misconduct in that aviation personnel have no personal interest in causing aviation incidents, while scientific misconduct may have a considerable payoff for those doing it, if it remains undetected (Eriksson and Helgesson 2013, p. 54). However, one should be aware that the criminalization of scientific misconduct may make researchers less likely to report incidence of misconduct, and hence lead to less detection of fraud, in comparison to a deviation report system. This is because the criminalization may raise the stakes and deter potential whistleblowers from reporting, as they may not want to send a colleague to jail or do not want to be involved in a criminal investigation.

For the sake of the argument, we will accept that there is a sound moral basis for criminalizing serious scientific misconduct, at least *prima facie*. In the subsequent sections we wish to point to some problems and complications that follow from such a position. In doing so, we start by raising some problems associated with equalizing serious scientific misconduct with FFP. We then argue that there is a risk associated with criminalizing misconduct in terms of its possible perceptual effects, as it may give researchers the false impression that instances of misconduct that falls outside the legal regulation do not count. Last, we suggest that if serious misconduct is not to be conflated with FFP, the demarcation issue largely remains. We also raise doubts about whether criminalizing the most serious forms of misconduct will lower the burdens for the universities or whether it will ensure fairness or help promote integrity in research.

## Difficulties with criminalizing FFP

Two important questions to ask when criminalizing any area are: Is the appropriate set of cases included, and is the appropriate set of cases excluded? Put differently, are any cases included that do not belong, and are there other cases that are excluded that should not be? If you want to criminalize serious cases of scientific misconduct, then your criteria should ideally include *all* serious cases and *nothing but* serious cases.

Nicholas Steneck (2006, p. 54) notes that it is commonly assumed that fabrication, falsification, and plagiarism are “the worst behaviors” in the area of scientific misconduct and deviations from good research practice. These are not the only forms of misconduct in research, of course. However, even though the definition of scientific misconduct varies between different jurisdictions, and in some cases even between universities (Resnik et al. 2015a, b), fabrication, falsification and plagiarism are indeed considered the paradigmatic examples of serious forms of misconduct. In line with this, some proponents of criminalizing scientific misconduct, such as Sovacool (2005), explicitly limit the concern to fabrication, falsification and plagiarism, while excluding other types of behavior. Sovacool holds that criminalization should only cover those who “purposely, knowingly, or recklessly commit acts of misconduct” (2005, p. 4). At the same time Sovacool explicitly states that these “would not include the comparatively minor offenses such as selective publishing of results, sins of omissions, sloppiness, inappropriate use of statistics, and dual submission of the same article to different journals” (ibid., p. 4). In what follows we criticize this proposal on the grounds that it includes too much, but also that it is too exclusive.

### Incorrect inclusions?

Our first criticism regarding equalizing FFP with serious scientific misconduct is that too much is included; that is, FFP contains cases that are not cases of serious scientific misconduct, or at least not obviously so. Some forms of plagiarism are an obvious case in point. Let us recall that plagiarism can be defined as “someone using someone else’s intellectual product (such as texts, ideas, or results), thereby implying that it is their own” (Helgesson and Eriksson 2015, p. 94). Plagiarizing groundbreaking ideas or large chunks of other researchers’ results are obvious examples of serious scientific misconduct while, at the opposite end of the scale, free-riding on the phrasing of a couple of sentences by copying and pasting from a fairly standard background or methods section is not—what harm does it do, apart from upsetting those emotionally engaged in battling plagiarism? (Helgesson 2015) In other words, plagiarism in research seems to vary considerably in terms of seriousness, ranging from clear cases of scientific misconduct to fairly insignificant deviations from good research practice.

Falsification is a very broad category. While it contains cases of extensive manipulation of data, it may also contain less serious cases. For instance, consider a study where four outliers have been eliminated when analyzing data; while it is argued in the paper that this is done based on specific theoretical considerations, mentioned in the paper, these as a matter of fact apply only to three of the four deleted measurement points, while there is no clear support for eliminating the fourth one. Another example of a less serious case could be that the research methods are described in a way that is not entirely correct, but the difference between what is described and what actually happened is slight and only makes limited difference to the outcome of the experiment.

Even fabrication could be argued to contain less serious cases, depending on what the judgment regarding seriousness is based on. If interpreted as serious deviation from good research practice—i.e., serious from a scientific perspective—then all fabrication is very serious. Arguably this should be enough as a basis for criminalization of scientific misconduct, if such criminalization is justified at all. But looking at societal consequences, the seriousness might be different. For instance, if someone fabricates data regarding the chemical composition of a comet passing at a safe distance from Earth, this may have little consequence outside academia (apart from the risk of contributing to the loss of trust in research if attracting a lot of media attention).

What this discussion clearly suggests is that it has to be settled whether seriousness as a basis for criminalization of scientific misconduct should strictly concern the degree of deviation from good scientific practice or if impact on health, wellbeing, safety, or social stability, or ecological or other environmental effects, should also be considered, and in that case how. Given how many of those arguing in favor of criminalization of scientific misconduct both consider FFP to be serious instances of misconduct while at the same appeal to the harm it may cause to the health and wellbeing of people as a reason for criminalization (see e.g., Redman and Caplan 2005; Sovacool 2005; Bhutta and Crane 2014), the fact that the connection between FFP and these sorts of harm is far from obvious in many cases should be acknowledged.

One might respond to our argument so far that although there are instances of FFP that arguably are not serious enough to warrant criminal punishment, the claim is not that all instances of FFP must be punished. Instead, by targeting FFP one will capture the most serious forms of fraud, although not all instances of FFP will therefore be punishable, if they are negligible. However, the claim that FFP is what ought to be criminalized is problematic also for the reason that drawing the line here excludes equally serious forms of misconduct.

### Incorrect exclusions?

Our second criticism regarding equalizing FFP with serious scientific misconduct is that important cases get excluded. There are other cases of deviation from good research practice that are as serious as FFP. We propose that withholding results may be as serious, in some cases. One example we have in mind is withholding results showing that a new medical drug, about to become accepted by national medical agencies such as the Food and Drug Administration (FDA) in the U.S. or Läkemedelsverket in Sweden, in fact has severe negative side effects potentially leading to death or substantial loss of health and wellbeing for those treated with it. Scientifically, selective publication may promote a highly misleading impression of the effects and efficacy of introducing a new drug, thus constituting a severely biased set of information upon which to base health care decisions.

It has also been pointed out that selective publication may be an even more worrisome factor for the possibility to reproduce scientific studies when compared to instances of falsification and fabrication (Pickett and Roche 2018). After having investigated public opinion on data fraud and selective publishing in the US, Pickett and Roche (2018) concluded that “there is a strong consensus among community members that both data fraud and selective reporting are morally wrong and deserving of serious sanctions.” Thus, contra Sovacool (2005), their results suggest that selective publishing of results is not conceived of as a minor offense. Given how it gives rise to similar problems of publications being misleading as falsification and fabrication, this is perhaps not so surprising. We suggest that selective publication is wrongful when misleading and sometimes threatens to cause non-trivial harm.

Focusing only on the impact on research integrity, there are other examples of deviations from good scientific practice that are as bad as or on par with FFP. For example, exaggerating the relevance and importance of one’s scientific results or presenting speculations as facts all have negative impact on the integrity of research and may be deeply misleading (Redman and Caplan 2005).

To make things even more complicated, another kind of case can be made for the position that criminalization of FFP alone would involve an unfortunate and mistaken exclusion of cases that actually should be included. The idea is that if the point of criminalizing scientific misconduct is to eliminate or reduce harm stemming from scientific research, whether internal to science or external to society, then it can be debated whether “serious harm” should be restricted to cases involving serious harm in the individual case or if focus should be on the kinds of cases that bring the greatest overall harm. Although most universities have their own cases of fabrication, falsification and plagiarism to feel ashamed of, evidence suggests that the prevalence is nevertheless fairly low, although numbers vary (Fanelli 2009; George 2016). In contrast, there may be other deviations from good research practice, perhaps harmless enough not even to be considered instances of scientific misconduct by some, but that are much more frequent and therefore likely to involve a greater amount of harm overall (Eriksson and Helgesson 2013; Zigmond and Fischer 2002). It may be the case that the widespread tendency to make one’s research results look a little bit better than they in fact are on the collective level leads to overconfidence in present beliefs and trends in results, thereby making it take longer before present positions and beliefs are questioned, thus slowing down progress in science. We suggest that this aspect is relevant to the ongoing debate on the difficulties to replicate results (Schmidt 2009; Francis 2012; Open Science Collaboration 2015; Anderson et al. 2016). As Zigmond and Fischer (2002) argue, the results section is “a prime area for misdemeanors” (p. 232). For example, common phrases such as “data not shown” or “unpublished observations” may be part of an effort to conserve space, but it could also be an attempt to influence the reader with data that could not stand up for scrutiny (Zigmond and Fischer 2002). Although it is arguably not on par with falsification or fabrication, this is a form of scientific misconduct which misleads the reader and may have a negative impact on research integrity. Yet, it cannot be excluded that these and other examples have a greater overall effect on scientific development than the total number of occurrences of fabrication, falsification and plagiarism. We don’t have the evidence saying that it does, but we here want to point at the difficulty in estimating what is worse: a widespread occurrence of minor deviations from good research practice or a limited set of occurrences of a much more serious kind. More importantly, as we discuss in greater detail below, this is something that will not be solved merely by criminalizing the most serious forms of scientific misconduct—this may even make the situation worse.

### Perceptual effects of drawing the line at FFP (or elsewhere)

One risk associated with criminalizing FFP, and leaving other deviations from good research practice outside the legal domain, is that such a choice may influence the perception of the instances falling outside the law. For instance, those already reluctant to see that there is anything wrong with practices deviating from proper scientific standards but falling outside FFP may draw the conclusion that one is now free to continue with these practices since they “clearly do not count”. This effect of stressing some unethical aspects of scientific practice and disregarding others occurs, or may occur, any time when there is legal regulation of an area of ethical significance. Hence, legally regulated ethical review focuses on some ethical aspects of studies, because the law says it should, and disregards others, because they are legally insignificant. Without further measures in order to counter less serious forms of scientific misconduct, this may be worrisome. As Zigmond and Fischer (2002) point out, inattention to minor and less serious misdemeanors “may communicate the wrong message about the value of responsible conduct to our community (i.e., the scientific community) and to the public-at-large” (p. 233).

Unlike our arguments so far, this concern is not necessarily limited to the case of criminalizing merely or primarily FFP. Rather, it is problematic at any point where there is a law which only targets a limited, yet serious set of scientific misconduct. With regards to the aims that a criminalization of scientific misconduct is held to serve, perceptual effects of this kind may be worrisome both for those who wish to increase integrity in research, but also for those who hold that criminalization is required for the sake of expressing appropriate moral blame or public disapproval, since the message that follows from punishing only certain types of misconduct will be a misdirected or perhaps even an dishonest message, especially if cases that are just as bad as FFP are excluded.

## Further complications from criminalizing scientific misconduct

Above we have highlighted a number of problems relating to criminalization of scientific misconduct with exclusive or primary focus on FFP. In this section, we argue that there are more general problems regarding criminalization of scientific misconduct. In particular, there will be demarcation issues as long as not all deviations from good research practice are criminalized. This, in turn, will be relevant to issues of efficiency, due process, and fairness.

### How should we draw the line?

One benefit with the suggestion that nothing but FFP should be criminalized is how it clearly indicates what should fall within the law and what should not. But since “serious misconduct” is not to be conflated with FFP, as we have argued, the challenge remains how to demarcate and how define the types of misconduct that should be criminal. How to do this exactly is beyond the scope of this paper, and partly something that should be left to legal expertise, namely the transferal of the normative position on scientific misconduct to the fabric of national or international law. One thing worth mentioning, however, is that when addressing this challenge it is not enough to simply claim that criminalization should be limited to deliberate fraud and misconduct, as proponents of criminalizing scientific misconduct sometimes argue (see e.g., Bhutta and Crane 2014). After all, there are forms of misconduct that, even if deliberate, are arguably not serious enough to be criminal. Similarly, grave negligence may also render some forms of misconduct serious enough to be appropriate targets of the law. The question therefore remains how to decide what ought to be considered serious enough, and what should not.

### The practical problem for the universities to a large extent remain

Unless the perceptual effects of drawing the line between criminal and non-criminal deviations from good research practice are that practices falling outside the legal regulation become perceived as unimportant and therefore are disregarded, the practical problem for the universities of handling cases of unethical scientific practice remains. While the law will handle some cases, many other cases will not be so handled and will therefore need to be taken care of by the universities themselves. This aspect is important to consider, especially given how criminalization of scientific misconduct is held to promote non-punitive ends as well. As we have pointed out previously, some of those defending criminalization of scientific misconduct have argued that the basic due process that would follow from criminalization is necessary in order to ensure a fair and balanced misconduct investigation (Sovacool 2005; Redman and Caplan 2005). It can also take away the burden associated with such investigations from the universities, and can help increase the efficiency of such investigations (Sovacool 2005). To what extent it really would grant balanced investigations, ensure fairness and increase efficiency, and as a result lower the burdens for the universities, would depend on what proportion of instances fall outside the legal regulation, and, for efficiency and relief of burden, on how time-consuming the sorting of cases to either category would be. If the law only targets the most serious examples of unethical research practices (regardless of if it only includes serious instances of FFP or is more inclusive) one can suspect that the majority of cases will still be handled by the universities and not by criminal courts. If so, it can be questioned whether such a system would be clearly more efficient, or fair, than one where universities handle all investigations into scientific misconduct.

In response, one should recognize that the criminalization still unburdens the universities in the sense that it makes it possible for them to focus more of their attention on the (individually) less serious types of misconduct. It would indeed be valuable if universities could focus more on the minor misdemeanors, which, as we have argued here, may be as problematic for research integrity overall as the most serious forms of scientific fraud. Taking minor misdemeanors seriously is important also in order to avoid the aforementioned perceptual effects that non-criminalized acts are unimportant.

Part of the work needed to be done regards prevention, by looking at research environment and culture, i.e., the breeding ground for unhealthy research practices. What are the research practices developed and maintained in different research groups and departments, and how do they relate to financial and other incentives? Are there ways to change incentives in ways that better align with the overall aims of research while fostering a more healthy research climate? The present stress on competition and achievements to the benefit of the individual researcher over teamwork and collective achievements to the benefit of mankind is problematic (Casadevall and Fang 2012), but not easily overcome.

Although this discussion is brief, it suggests that criminalization will not be the solution to the problems facing universities with regards to the prevalence of scientific misconduct, nor will it necessarily increase integrity in research. To this end, far more is required. The urge for criminalization, as it has been defended in the bioethics literature, is an indication of how hard it is to create an environment with clear incentives for individual researchers to also abide with good scientific conduct—as the need for criminalization is defended on the grounds that it will act as a deterrent where there currently are no or few disincentives. However, unlike those overly optimistic about the anticipated effects of criminalization, we believe that criminalization will not in itself bring about all the effects that proponents have expected.

## Concluding remarks

This paper has discussed criminalization of scientific misconduct, as it has been discussed and defended in the bioethics literature. It has been argued that one can make a case in favor of criminalizing scientific misconduct, the most obvious benefits being offensive measures to combat harmful wrongdoing in research and promotion of due and fair process in investigations into suspected misconduct. However, the benefits may not be as great as criminalization proponents seem to have hoped for, since criminalization of some acts of scientific misconduct will leave many other acts to still be dealt with by individual universities. Without further measures one is at risk of giving the false impression that dubious practices falling outside the legal regulation “do not count”, which can have negative impact on research integrity. We have also argued that restricting criminalization to fabrication, falsification, and plagiarism would be a mistake since FFP does not identify the relevant set of serious cases of scientific misconduct—it fails both in terms of incorrect inclusions and incorrect exclusions. Also, as long as not all deviations from good research practice are criminalized there will be important demarcation issues which proponents of criminalization sometimes overlook.
